# Relationship Between Nutritional Status and Clinical Outcome in Patients With Gastrointestinal Stromal Tumor After Surgical Resection

**DOI:** 10.3389/fnut.2022.818246

**Published:** 2022-02-02

**Authors:** Ping'an Ding, Honghai Guo, Chenyu Sun, Peigang Yang, Yuan Tian, Yang Liu, Zhidong Zhang, Dong Wang, Xuefeng Zhao, Bibo Tan, Yu Liu, Yong Li, Qun Zhao

**Affiliations:** ^1^The Third Department of Surgery, The Fourth Hospital of Hebei Medical University, Shijiazhuang, China; ^2^AMITA Health Saint Joseph Hospital Chicago, Chicago, IL, United States

**Keywords:** gastrointestinal stromal tumors, surgical resection, nutrition status, NRS2002, PG-SGA

## Abstract

**Background:**

Currently, gastrointestinal stromal tumors (GIST) are the most common mesenchymal tumors in the gastrointestinal tract, and surgical resection is the main treatment. Malnutrition after gastrointestinal surgery is not uncommon, which may have adverse effects on postoperative recovery and prognosis. However, the nutritional status of GIST patients after surgical resection and its impact on clinical outcomes have received less attention. Therefore, the aim of this study was to dynamically evaluate the nutritional status of GIST patients undergoing surgical resection, and to analyze the correlation between nutritional status and clinical outcomes.

**Methods:**

We retrospectively analyzed the clinical data of GIST patients who underwent surgical resection in the Fourth Hospital of Hebei Medical University from January 2016 to January 2020. Nutritional risk screening 2002 (NRS2002) and Patient-Generated Subjective Global Assessment (PG-SGA) were used to assess the nutritional status of all patients at admission and discharge, and the correlation between nutritional risk and clinical outcomes was analyzed.

**Results:**

A total of 413 GIST patients were included in this study, among which 114 patients had malnutrition risk at admission (NRS2002 score ≥ 3), and 65 patients had malnutrition (PG-SGA score ≥ 4). The malnutrition risk rate (27.60 vs. 46.73%, *p* < 0.001) and malnutrition incidence (15.73 vs. 37.29%, *p* < 0.001) at admission were lower than those at discharge. Compared with the laboratory results at admission, the albumin, prealbumin, and total protein of the patients at discharge were significantly lower (all *p* < 0.05). And there was a negative correlation between PG-SGA and clinical outcome (all *p* < 0.05).

**Conclusion:**

The nutritional status of GIST patients after surgical resection at discharge was worse than that at admission, and malnutrition is an important risk factor leading to poor clinical outcomes.

## Introduction

Presently, gastrointestinal stromal tumors (GIST) are increasing rapidly worldwide, mostly due to mutations in KIT and PDGFRA genes (1, 2). In recent years, with the advancement of molecular biology research on GIST, the treatment mode has made breakthrough progress, but surgical resection is still the mainstay and most effective treatment for GIST (3–5). However, some patients experienced nutritional deterioration after surgery, especially in patients undergoing gastrointestinal resection, which often resulted in reduced food intake and weight loss. Numerous studies have demonstrated that malnutrition is consistently associated with negative outcomes, such as high perioperative infection rates, long hospitalization time, and short survival time (6–8).

However, until now, numerous studies focused on the nutritional status of patients such as gastric cancer and other malignant cancer, while few people pay attention to the nutritional status of GIST patients, especially those with surgical resection at discharge (9–11). Our previous study has found that 77.76% of newly diagnosed GISTs were at risk of malnutrition (Nutritional risk screening 2002 score ≥ 3), and the incidence of malnutrition was 10.09% (Patient-Generated Subjective Global Assessment score ≥ 4) at admission (12). This suggests that malnutrition is common in newly diagnosed GIST patients on admission. Similarly, the nutritional status assessment of GIST patients after surgical resection at discharge is equally important and requires attention. Meanwhile, there are a large number of published studies showing that malnutrition in surgical patients after discharge, which will affect the quality of life of patients and lead to delayed postoperative treatment and increased mortality (13–15). However, the relationship between nutritional status and clinical outcomes of GIST patients after surgical resection is also unclear. Therefore, attention to malnutrition in GIST patients will be particularly important for improving the quality of life and significantly prolonging the survival period.

Currently, many nutritional guidelines recommend standardized nutritional supports, including nutritional screening, assessment, intervention, and monitoring. Among them, the nutritional screening is the first step, and NRS2002 is the recommended screening tool (16–22). Meanwhile, PG-SGA is a nutritional assessment method developed on the basis of Subjective Global Assessment (SGA) designed specifically for cancer patients (23). It has been confirmed by clinical studies in various countries that it can be used for nutritional assessment of tumor patients and is an effective tool for evaluating the specific nutritional status of tumor patients (24). In addition, the physical measurement indexes including body weight, body mass index (BMI), and grip strength, as well as blood biochemical parameters including lymphocytes, albumin, and prealbumin, are also commonly used to evaluate the nutritional status of patients (25, 26).

At present, there is no standard nutritional evaluation method for GIST patients undergoing surgical resection, and there is no consensus on which evaluation method will be the best choice. Moreover, there are few studies on the application of NRS2002 combined with PG-SGA in the perioperative assessment of GIST patients. Therefore, in the present study, we used NRS2002 combined with PG-SGA and other nutritional indicators to evaluate the nutritional status of patients with GIST after surgical resection, in order to clarify whether postoperative nutritional status is related to adverse clinical outcomes.

## Methods and Materials

### Patient Section

This study retrospectively analyzed the medical data of 413 GIST patients who underwent surgical resection in our hospital from January 2016 to January 2020. Inclusion criteria were as follows: (1) pathological diagnosis was GIST; (2) radical surgical resection; (3) without preoperative anti-tumor treatment; (4) completion of Quality of Life Questionnaire; and (5) detailed and complete clinical data. Exclusion criteria were as follows: (1) the patient had accepted antitumor therapies before surgery; (2) patients with cognitive impairment or other acute psychological problems; (3) those without complete medical records and laboratory results; (4) inpatients who were admitted due to surgical emergency; (5) patients who refused to accept assessment or do not sign informed consent. All procedures performed in studies involving human participants were in accordance with the ethical standards of the institutional and/or national research committee and with the 1964 Declaration of Helsinki and its later amendments or comparable ethical standards. This study was tested and approved by the ethics committee of The Fourth Hospital of Hebei Medical University, and the patients provided informed consent.

### Assessment Method

All patients completed anthropometry, NRS2002 screening, PG-SGA assessment, and blood biochemical parameters examination within 24 h after admission and 24 h before discharge, and NRS2002 screening and PG-SGA evaluation were evaluated by the same group of physicians. The anthropometry of patients included weight, upper arm circumference, and grip strength. The blood biochemical parameters examination included serum hemoglobin, albumin, prealbumin, and total protein, etc. All patients were screened by NRS2002 score after admission, and the score was ≥3, indicating that there was a risk of malnutrition in patients. PG-SGA score includes patient self-assessment and medical staff assessment, which includes seven areas (27). Patients' self-assessment includes weight changes, dietary intake, eating symptoms, and physical activity and function. Medical staff assessment includes nutrition-related disease status, metabolic status, and physical examination. Each of these seven areas is given a score of 0–4, and the sum of scores obtained in each area is divided into quantitative and qualitative evaluations, thus providing guidance on the level of nutrition and drug intervention required by each patient. Quantitative evaluation results are as follows: PG-SGA score of 0–1 indicates that nutritional support not required and treatment in the future based on routine re-evaluation, 2–3 points indicate malnutrition or suspected malnutrition, 4–8 points indicate moderate malnutrition, and ≥9 points indicate severe malnutrition (27).

### Quality of Life Assessment

The quality of life of patients was assessed using the European Organization for Research and Treatment of Cancer Quality of Life Questionnaire Core 30 (EORTC-QLQ-C30) 3.0 version, which was composed of 30 items, including five functional scales (physical, role, emotional, cognitive and social functioning functions), three symptom scales (fatigue, nausea/vomiting, and pain), a global health status/quality of life domain and six single symptoms (dyspnea, insomnia, appetite loss, constipation, diarrhea, and financial problems) (28). The functional and symptom scale score was divided into four grades, and the direct score was 1 (no) to 4 (very). The global health scale score was divided into seven grades, and the score was 1 (very poor) to 7 (very good) according to the patient's response options. The mean value of each subscale was linearly converted to the range of 0–100 scores. Higher scores in global health status and functional scales indicate better quality of life, whereas higher scores in the symptom scales indicate more severe symptoms. In this study, all patients were investigated by EORTC-QLQ-C30 questionnaire 1 month after discharge.

### Clinicopathological Parameters and Definitions

We collected the basic data of newly diagnosed GIST patients including gender, age, weight, etc. Laboratory tests include routine blood tests and biochemical tests. Preoperative examination included abdominal computed tomography (CT), nuclear magnetic resonance imaging (MRI), and gastrointestinal endoscopy. Pathology and gene detection included tumor location, tumor size, mitotic count, immunohistochemistry, risk classification, c-kit exons 9, 11, 13 and 17, and PDGFRA exons 12 and 18. The risk classification standard we adopted is the 2008 version of the improved National Institutes of Health (NIH) classification (29).

Meanwhile, the clinical related outcome indicators were recorded, including hospitalization time, complications, and expenses. The hospitalized complications included infectious complications and other complications. Infectious complications are defined as the presence of pathogens in the body's original sterile tissues and confirmed by pathogen culture results, or there are clinical symptoms and signs, imaging or hematological evidence related to infection (30). The discharge standard is as follows: patients can live self-care, normal urination, normal body temperature, and no need for intravenous infusion (31).

### Statistical Analyses

All statistical analyses were performed using SPSS 21.0 soft-ware (IBM, Armonk, NY, USA) and GraphPad Prism 8.01 (GraphPad Software, San Diego, California). All continuous variables are tested for normal distribution by Kruskal-Wallis test. The variable of normal distribution is represented by mean ± standard deviation, and the variable of non-normal distribution is represented by median. The classification variables were compared by χ^2^ or Fisher exact test, and the continuous variables were compared by independent *t*-test or Mann-Whitney *U*-test. Logistic regression analysis was used for multivariate analysis of the risk of postoperative complications. According to the potential confounding factors, multiple linear regression analysis was used to evaluate the correlation between nutritional indicators (NRS2002, PG-SGA, weight, upper arm circumference, grip strength, serum hemoglobin, albumin, prealbumin, and total protein) and EORTC-QLQ-C30 scale. *P*-value < 0.05 was regarded as statistical difference significantly.

## Results

### Patient Characteristics

**Figure 1:**
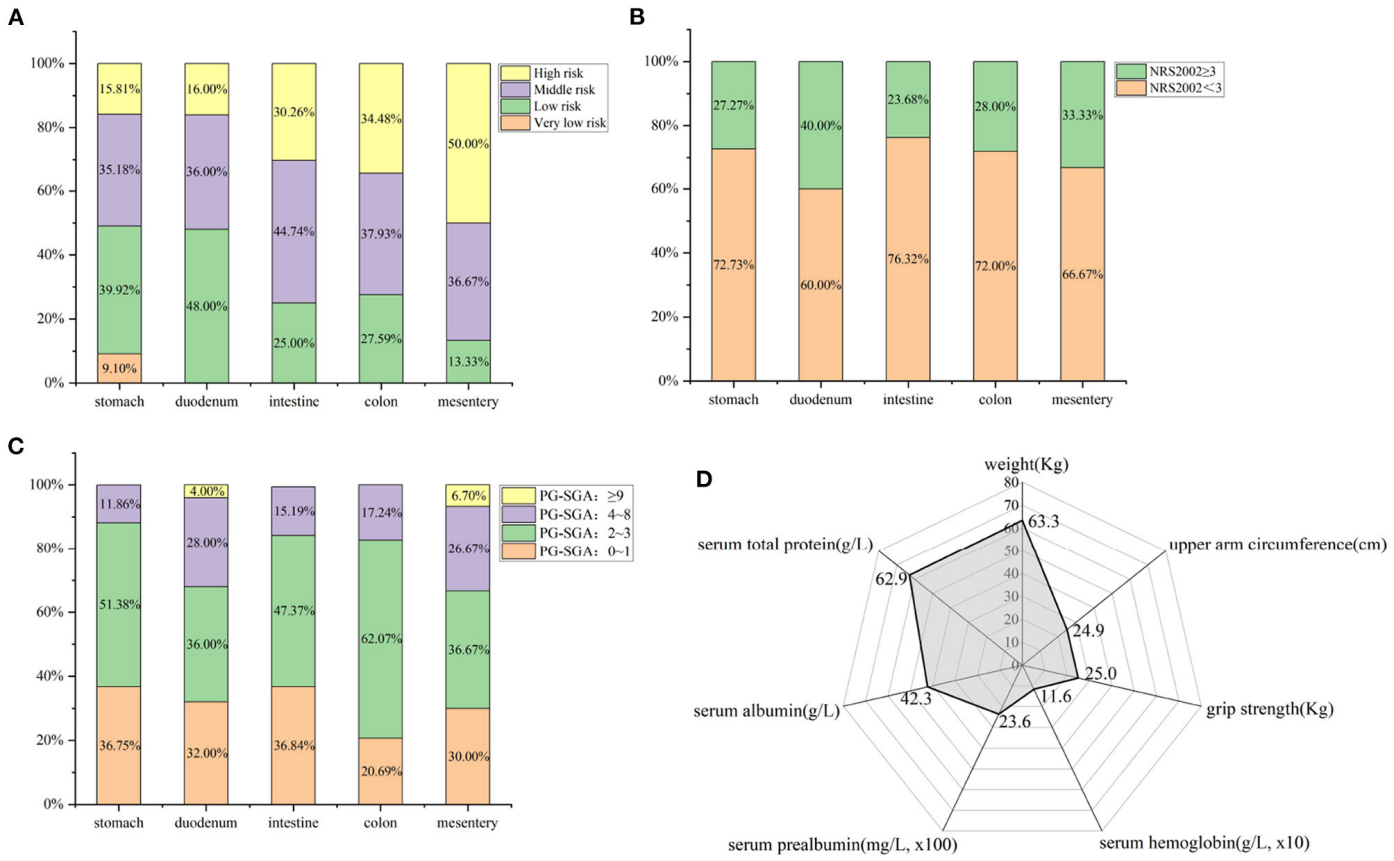
Baseline data of 413 GIST patients at admission. **(A)** Disease distribution according to different tumors; **(B)** NRS2002 nutritional risk screening; **(C)** PG-SGA nutritional assessment; **(D)** Other nutrition-related indicators (Mean).

Between January 2016 to January 2020, 413 GIST patients were screened for inclusion. According to the 2008 version of NIH stromal tumor risk classification standard, 92 cases (22.28%) of high risk group, tumor location is mostly mesentery; there were 154 cases (37.29%) in the middle risk group, and 144 cases (34.86%) in the low risk group, while 23 cases (5.57%) in the very low risk group were all located in the stomach (Figure 1A). All GIST patients were confirmed by pathology and underwent R0 resection, including 23 cases of combined organ resection. Among these 23 patients, there were 8 cases of combined splenectomy, 6 cases of partial hepatectomy, 5 cases of pancreatic tail resection, 2 cases of cholecystectomy, 1 case of oophorectomy, and 1 case of partial bladder resection. After surgical resection, 413 patients with postoperative pathology of high-risk group (22.28%) and medium-risk group (37.29%) were treated with oral targeted drug imatinib, while patients in low-risk group and extremely low-risk group were regularly reviewed. Other baseline demographic and clinical features of the whole group are shown in Table 1.

**Table 1:** Patient baseline demographic and clinical characteristics at admission.

**Variables**	***N*** **(Percentage)**
**Age (years)**	59.7 ± 10.3 *
**Sex (male)**	201 (48.32%)
**Tumor location**	
Stomach	253 (61.26%)
Duodenum	25 (6.05%)
Intestine	76 (18.40%)
Colon	29 (7.02%)
Mesentery	30 (7.26%)
**Tumor size (cm)**	5.3 ± 4.8*
**Nuclear mitotic figure (50HPF)**	
<5	149 (36.08%)
6~10	236 (57.14%)
>10	28 (6.78%)
**c-kit exons**	
Positive	268 (64.89%)
Negative	145 (35.11%)
**PDGFRA exons**	
Positive	112 (27.12%)
Negative	301 (72.88%)

* *Mean ± SD*.

All patients underwent NRS2002 screening and PG-SGA assessment at admission, and Figures 1B,C show the nutritional risk and assessment of 413 GIST patients on admission. Among them, 114 patients (27.60%) had the risk of malnutrition (NRS2002 score ≥ 3), and 65 patients (15.74%) had malnutrition (PG-SGA score ≥ 4). Meanwhile, the average weight of all patients at admission was 63.3 Kg, and the average grip strength was 25.0 Kg. The average values of laboratory-related nutritional indicators such as serum albumin, prealbumin and total protein were 42.3 g/L, 236.0 mg/L, and 62.9 g/L, respectively (Figure 1D).

### Changes of NRS2002 Score and PG-SGA Score at Admission and Discharge

**Figure 2:**
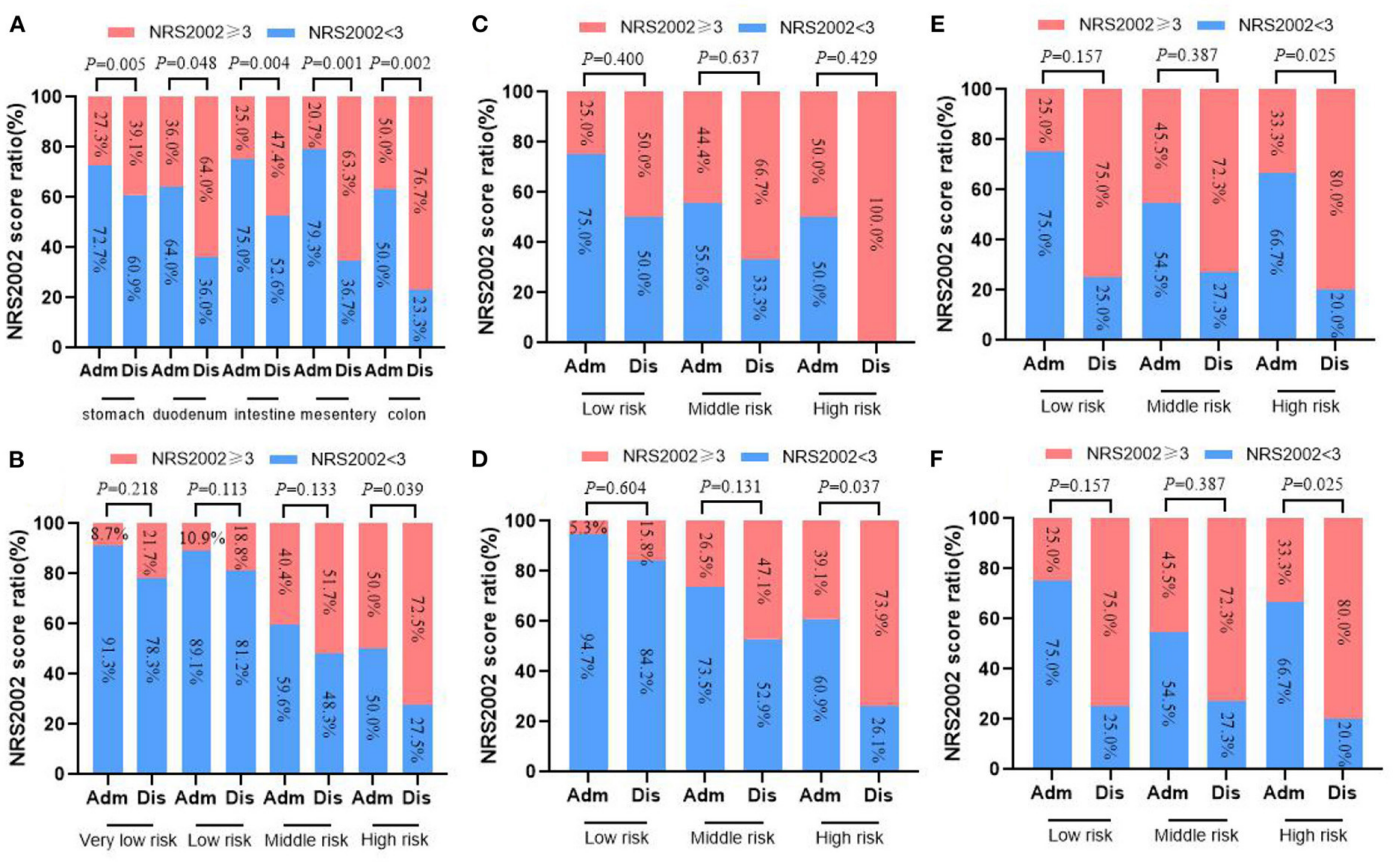
Changes of NRS2002 screening at admission and discharge in 413 GIST patients. **(A)** Total; **(B)** stomach; **(C)** duodenum; **(D)** intestine; **(E)** colorectal; **(F)** mesentery.

All patients completed NRS2002 screening and PG-SGA assessment at admission and upon discharge. At admission, 299 cases (72.40%) had NRS2002 score < 3, and 114 cases (27.60%) had NRS2002 score ≥3. However, 193 cases (46.73%) had nutritional risk at discharge (NRS2002 score ≥ 3), and the difference was statistically significant (*p* < 0.001). Meanwhile, based on the different tumor locations, the proportion of NRS2002 score ≥ 3 at discharge was also higher than that at admission (all *p* < 0.05). Moreover, for patients at the same tumor location, NRS2002 scores had no difference between admission and discharge for those with risk grade below middle risk (all *p* > 0.05), but the difference was significant only among high-risk patients (all *p* < 0.05) (Figure 2).

**Figure 3:**
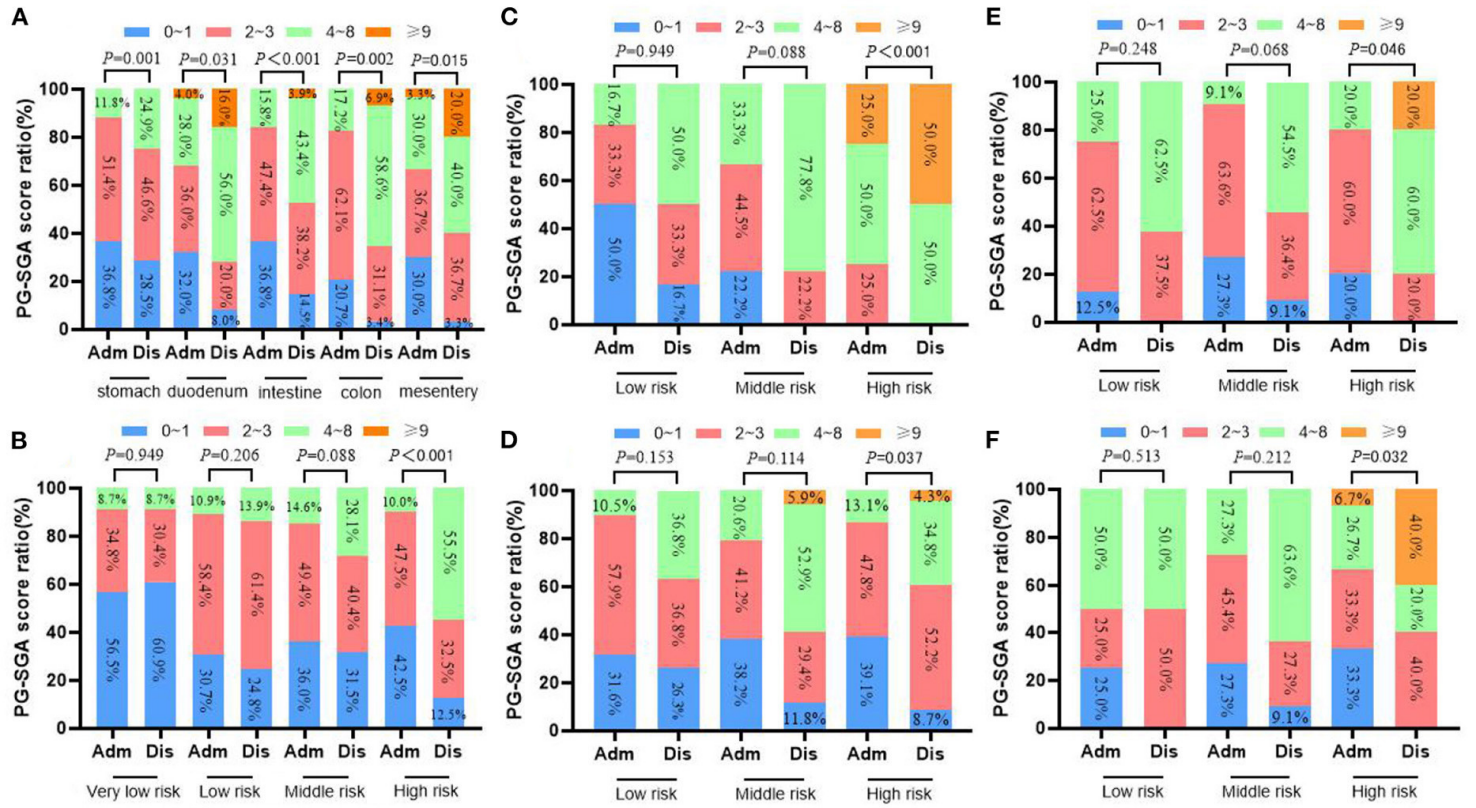
Changes of PG-SGA nutritional assessment in 413 GIST patients at admission and discharge. **(A)** Total; **(B)** stomach; **(C)** duodenum; **(D)** intestine; **(E)** colorectal; **(F)** mesentery.

Furthermore, only 65 patients had malnutrition at admission (PG-SGA score ≥ 4), while the proportion increased significantly at discharge (15.73 vs. 37.29%, *p* < 0.001). There were 154 patients with PG-SGA score ≥ 4 points at discharge, of which PG-SGA score ≥ 9 accounted for 3.15%. In addition, the number of patients at different tumor locations with PG-SGA score ≥ 4 at discharge was higher than at admission (all *p* < 0.05). Subgroup analysis of GIST patients at the same tumor site showed that especially for patients at high risk, they are more likely to suffer from malnutrition than before admission (PG-SGA score ≥ 4) (all *p* < 0.05) (Figure 3).

**Figure 4:**
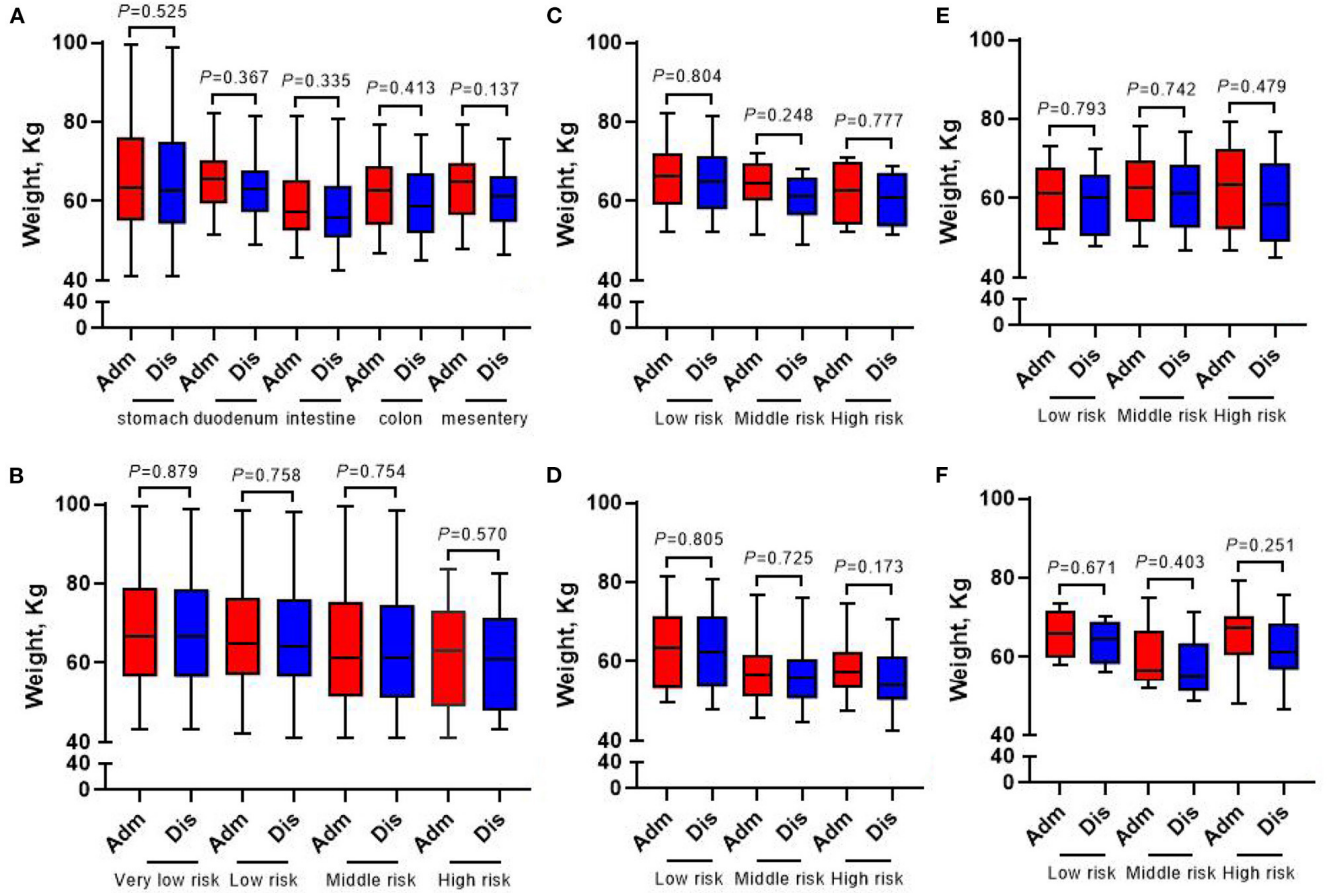
Changes of weight in 413 GIST patients at admission and discharge. **(A)** Total; **(B)** stomach; **(C)** duodenum; **(D)** intestine; **(E)** colorectal; **(F)** mesentery.

**Figure 5:**
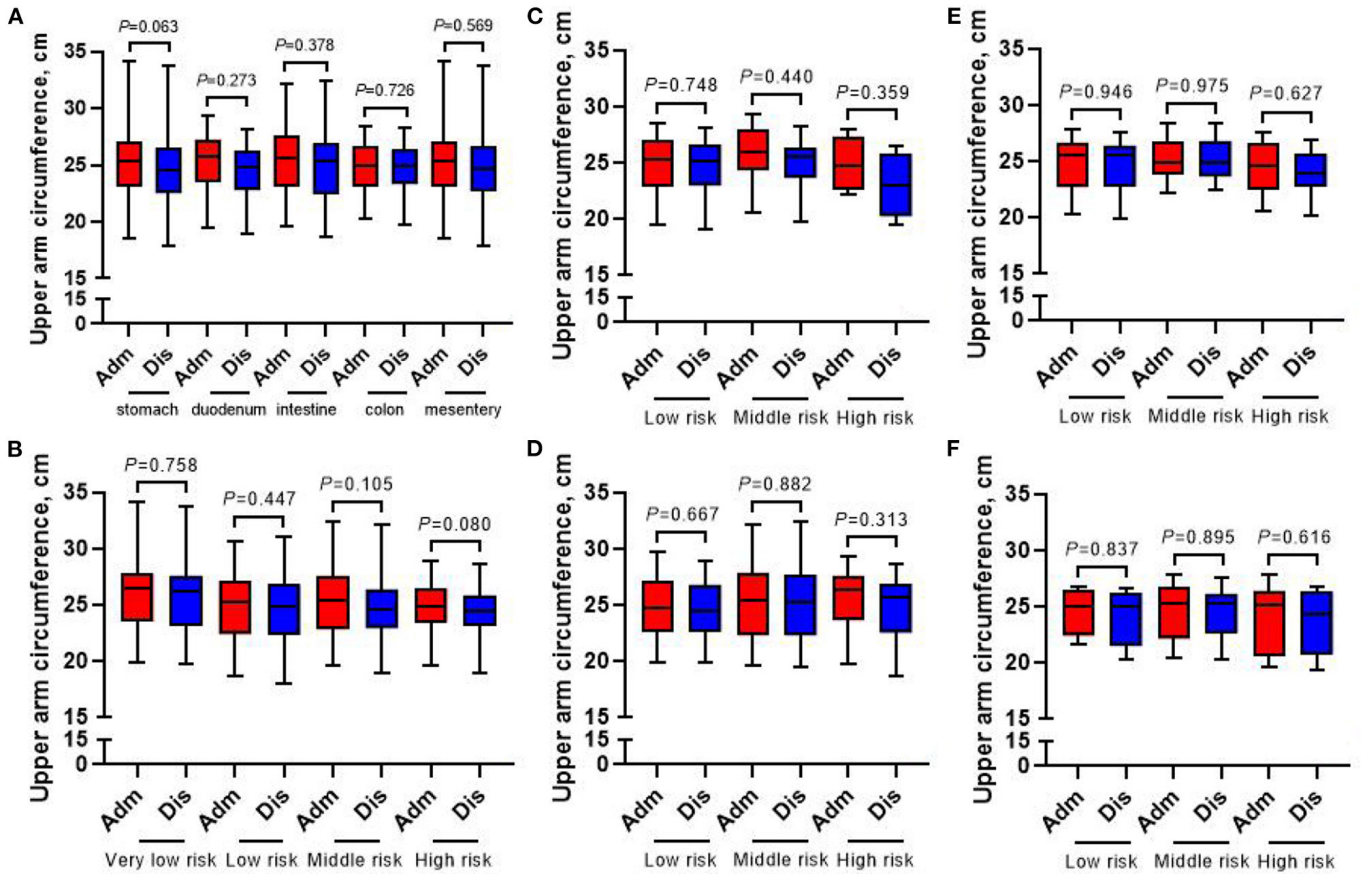
Changes of upper arm circumference in 413 GIST patients at admission and discharge. **(A)** Total; **(B)** stomach; **(C)** duodenum; **(D)** intestine; **(E)** colorectal; **(F)** mesentery.

**Figure 6:**
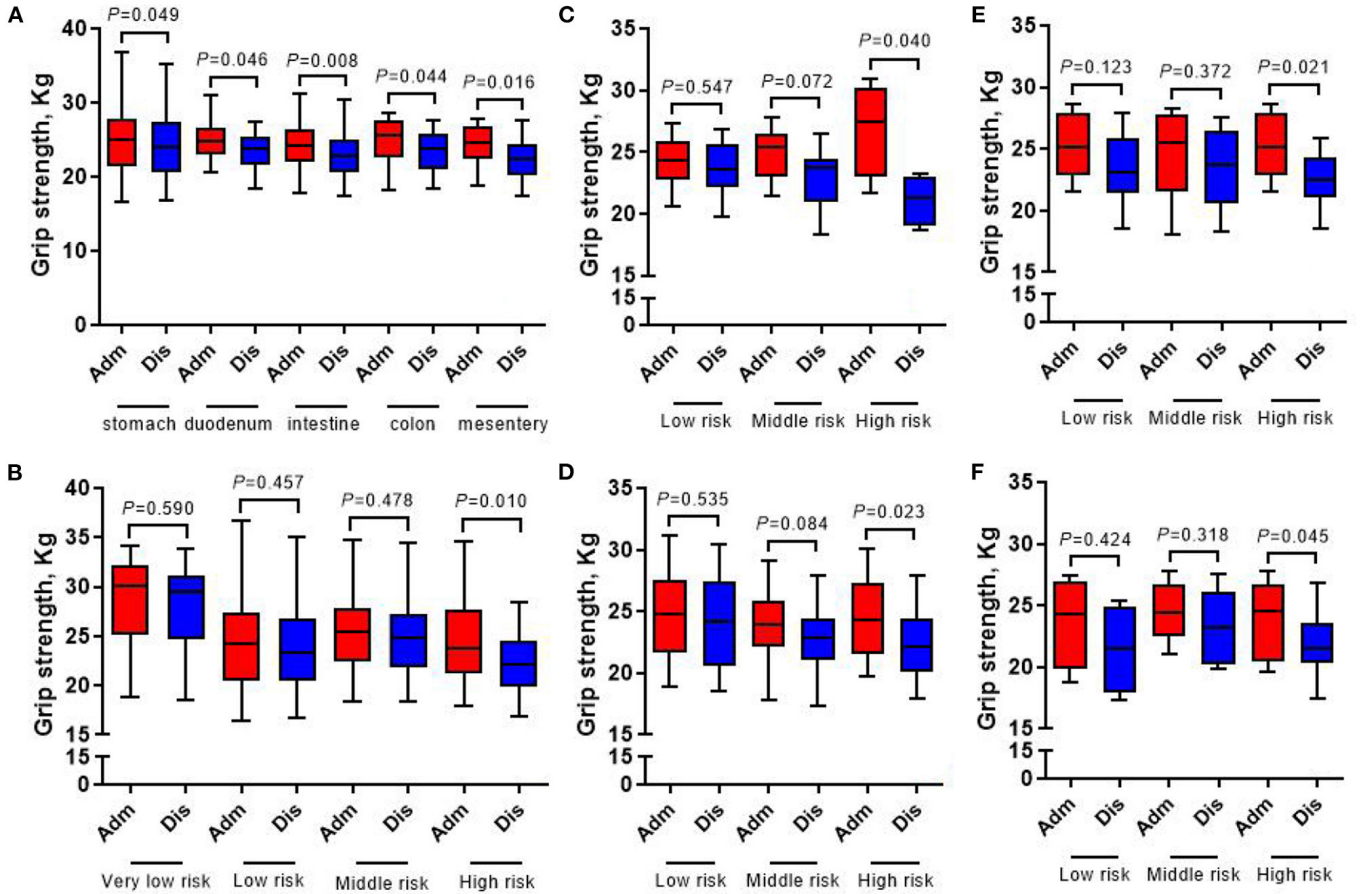
Changes of grip strength in 413 GIST patients at admission and discharge. **(A)** Total; **(B)** stomach; **(C)** duodenum; **(D)** intestine; **(E)** colorectal; **(F)** mesentery.

Figures 4, 5 show that compared with the time of admission, whether GIST patients located at different tumor sites or patients with different risk grades at the same tumor site, there was no difference in their body weight and upper arm circumference at the time of discharge (all *p* > 0.05). However, in terms of patient grip strength, we found that there were significant differences between GIST patients with different tumor sites at admission and at discharge. Further analysis showed that for patients with different risk grades, only high-risk GIST patients have such obvious differences (Figure 6).

### Changes of Laboratory Examination Indexes at Admission and Discharge

**Figure 7:**
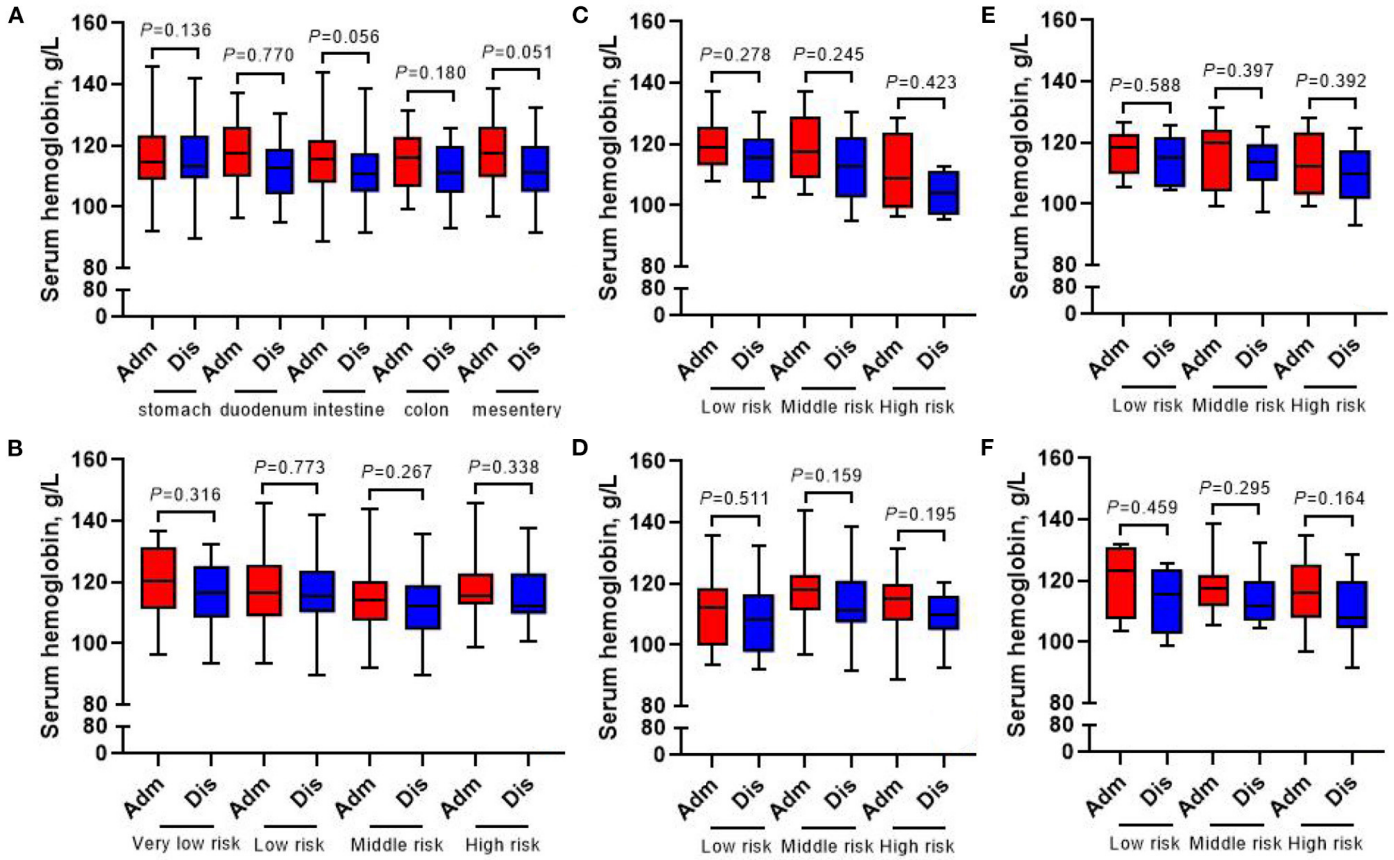
Changes of serum hemoglobin in 413 GIST patients at admission and discharge. **(A)** Total; **(B)** stomach; **(C)** duodenum; **(D)** intestine; **(E)** colorectal; **(F)** mesentery.

**Figure 8:**
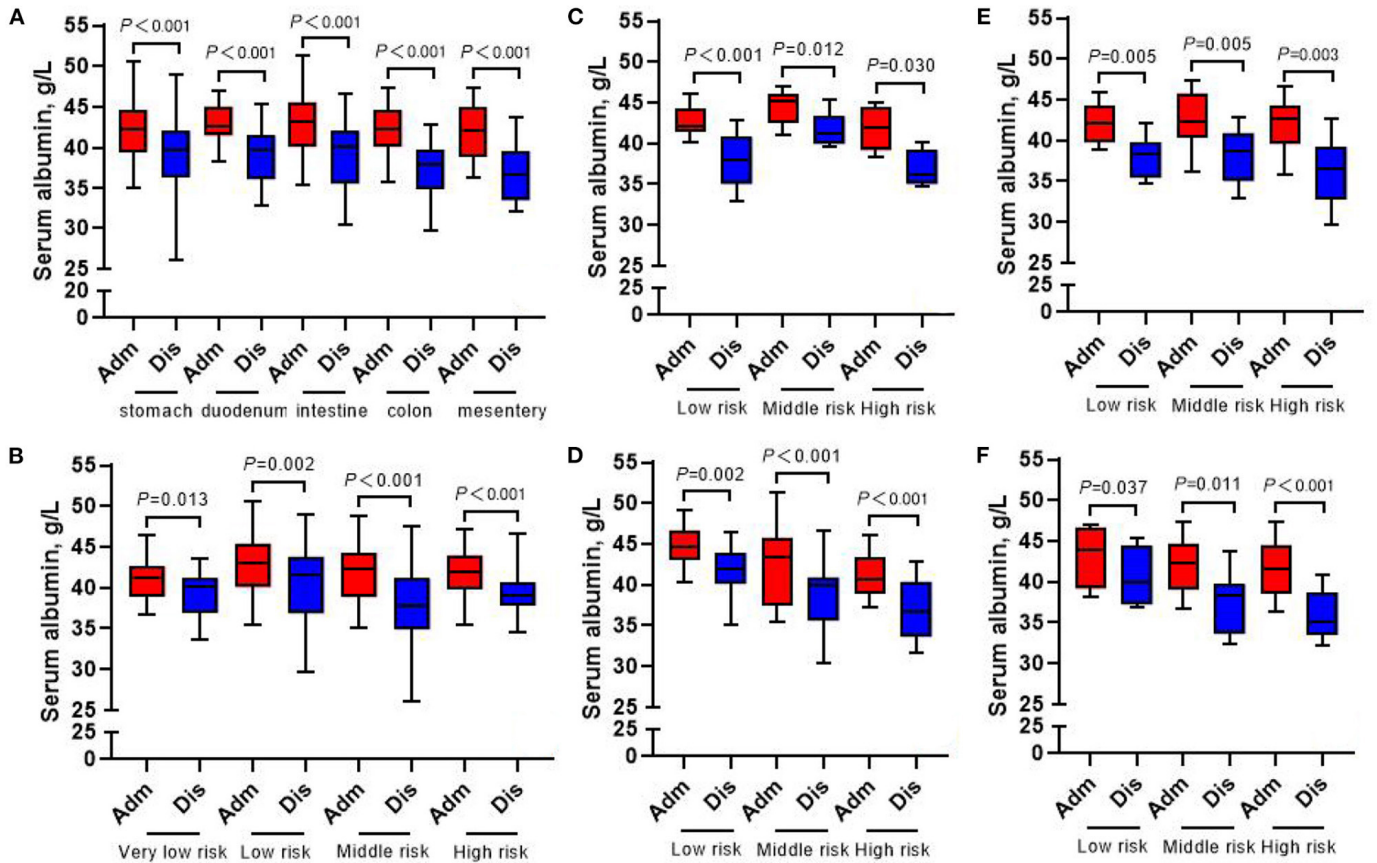
Changes of serum albumin in 413 GIST patients at admission and discharge. **(A)** Total; **(B)** stomach; **(C)** duodenum; **(D)** intestine; **(E)** colorectal; **(F)** mesentery.

**Figure 9:**
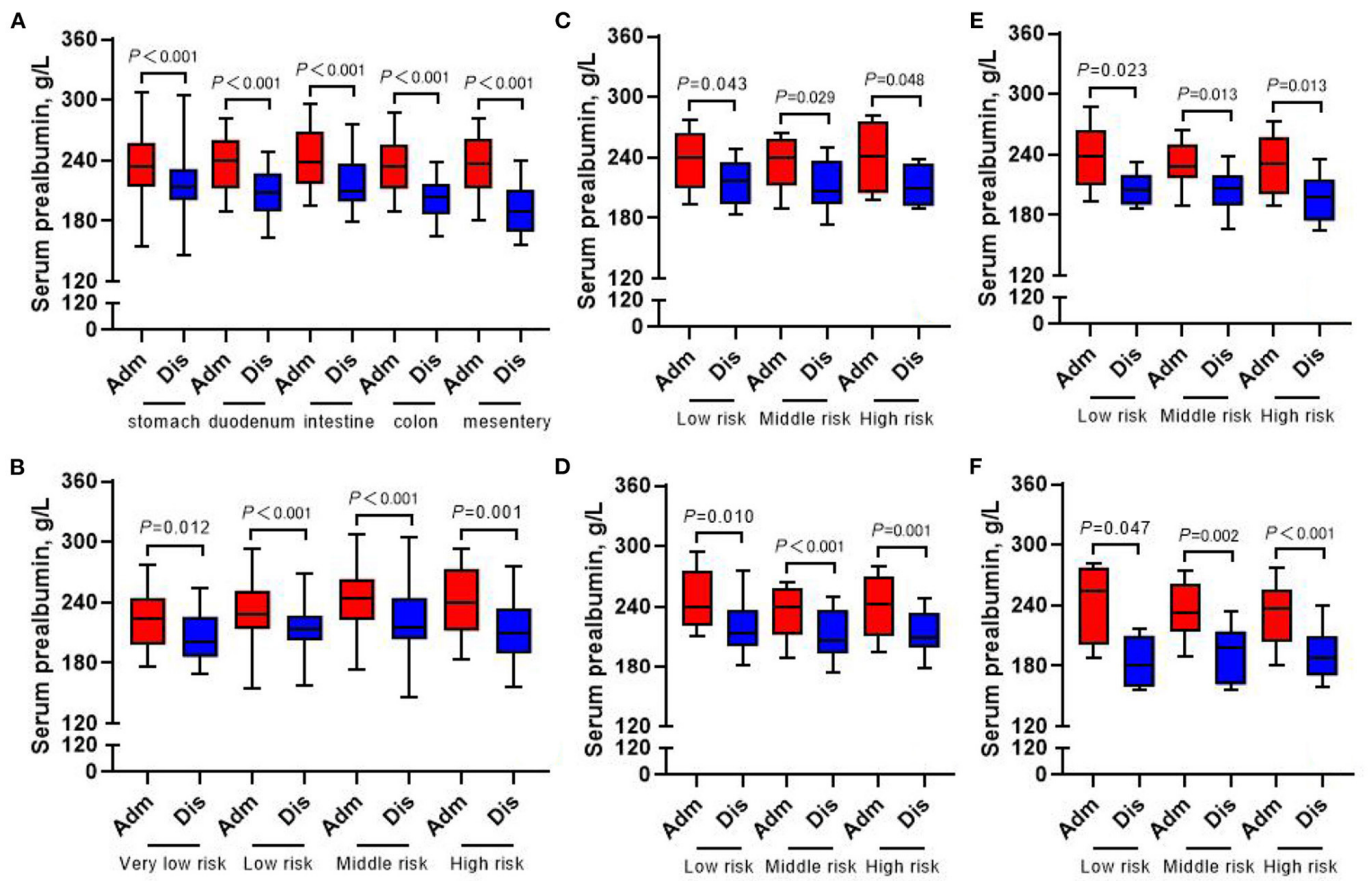
Changes of serum prealbumin in 413 GIST patients at admission and discharge. **(A)** Total; **(B)** stomach; **(C)** duodenum; **(D)** intestine; **(E)** colorectal; **(F)** mesentery.

**Figure 10:**
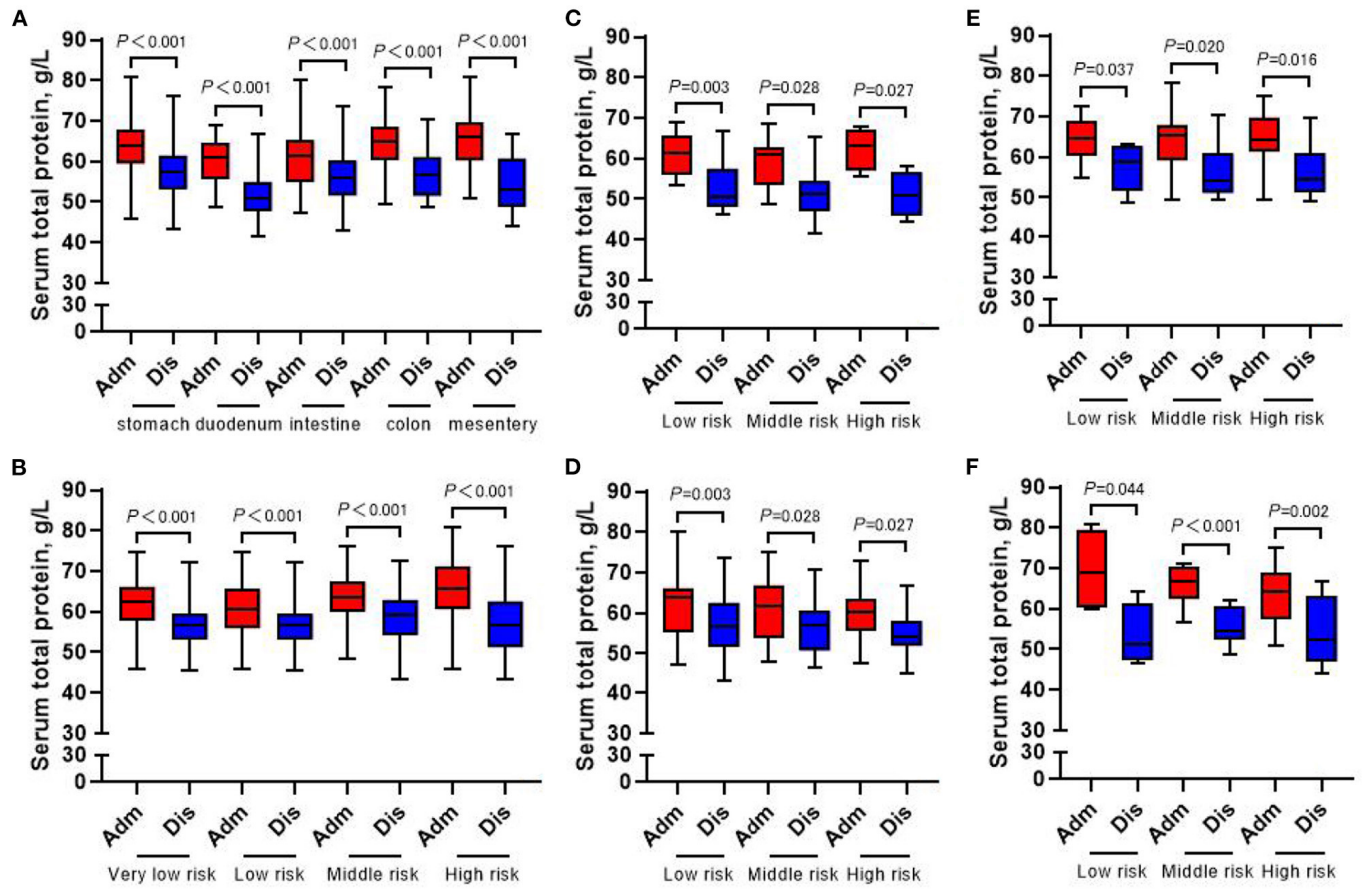
Changes of serum total protein in 413 GIST patients at admission and discharge. **(A)** Total; **(B)** stomach; **(C)** duodenum; **(D)** intestine; **(E)** colorectal; **(F)** mesentery.

The whole group of patients underwent nutrition-related peripheral blood laboratory tests at admission and at discharge, and the change in hemoglobin level was not statistically significant (all *p* > 0.05) (Figure 7). In terms of changes in other laboratory indicators, whether in accordance with the different tumor locations or the different risk grades for subgroup analysis, the albumin, prealbumin, and total protein of all patients at discharge were lower than those at admission (all *p* < 0.05) (Figures 8–10).

### Nutritional Support and Postoperative Complications

Analysis of nutritional support based on different PG-SGA scores showed that 65 patients needed nutritional intervention at admission (PG-SGA score ≥ 4). However, only 49 patients (75.38%) received nutritional support 1 week before treatment, of which 9.52% received parenteral nutrition (PN) support, 50.77% received enteral nutrition (EN) support, and 15.38% received both EN and PN support. In addition, we also found that the proportion of patients who needed nutritional intervention at discharge (PG-SGA score ≥ 4) was higher than that at admission, but only 62 cases (40.26%) received nutritional support at discharge (Table 2).

**Table 2:** Patient-generated subjective global assessment classification and nutritional support situation [*n* (%)].

**Nutrition support**	**Admission PG-SGA**				**Discharge PG-SGA**			
	**0~1** **(***N*** = 144)**	**2~3** **(***N*** = 204)**	**4~8** **(***N*** = 63)**	**≥9** **(***N*** = 2)**	**0~1** **(***N*** = 87)**	**2~3** **(***N*** = 172)**	**4~8** **(***N*** = 139)**	**≥9** **(***N*** = 15)**
No	140 (97.22)	193 (94.08)	16 (25.40)	0 (0)	80 (89.89)	151 (87.79)	87 (62.59)	5 (33.33)
Yes								
PN	0 (0)	1 (0.49)	6 (9.52)	0 (0)	0 (0)	0 (0)	0 (0)	0 (0)
EN	4 (2.78)	10 (4.90)	32 (50.79)	1 (50.00)	7 (10.11)	21 (12.21)	52 (37.41)	10 (66.67)
EN and PN	0 (0)	0 (0)	9 (14.29)	1 (50.00)	0 (0)	0 (0)	0 (0)	0 (0)

*PN, parenteral nutrition; EN, enteral nutrition; PG-SGA, patient-Generated Subjective Global Assessment*.

Among the 413 patients, 82 cases (19.85%) had postoperative complications, including 24 cases of surgical-related complications, 55 cases of respiratory complications, and 3 cases of cardiovascular complications. The patients were divided into two groups based on the PG-SGA score at admission. The incidence of complications in the PG-SGA ≥ 4 group was 29.23% (19/65), which was significantly higher than that in the PG-SGA <4 group (18.10%, 63/348) (*p* = 0.039). In order to reduce the interference of nutritional support on the incidence of postoperative complications, the comparison between group B and group D without nutritional support showed that the incidence of postoperative complications in the group without malnutrition (PG-SGA <4) was lower than that in the group with malnutrition (PG-SGA ≥ 4) (18.02 vs. 56.25%, *p* <0.001). Further subgroup analysis of group C and group D with simultaneous malnutrition (PG-SGA ≥ 4) showed that the incidence of postoperative complications in group C with preoperative nutritional support was significantly lower than that in group D without nutritional support (20.41 vs. 56.25%, *p* = 0.006, Table 3).

**Table 3:** Comparison of postoperative complications based on PG-SGA score [*n* (%)].

**Variable**	**PG-SGA <4**		***P***	**Total 1**	**PG-SGA** **≥4**		***P***	**Total 2**	***P*** *	***P*** **
	**Support (A)** **(***N*** = 15)**	**No support (B)** **(***N*** = 333)**			**Support (C)** **(***N*** = 49)**	**No support (D)** **(***N*** = 16)**				
Total	3 (20.00)	60 (18.02)	1.000^b^	63 (18.10)	10 (20.41)	9 (56.25)	0.006	19 (29.23)	<0.001	0.039
Wound infection	1 (6.67)	3 (0.90)	0.417^b^	4 (1.15)	1 (2.04)	1 (6.25)	0.990^b^	2 (3.08)	0.446^b^	0.530^b^
Anastomotic leakage	0 (0)	4 (1.20)	–	4 (1.15)	1 (2.04)	0 (0)	–	1 (1.54)	–	1.000^b^
Lymphatic leakage	0 (0)	0 (0)	–	0 (0)	0 (0)	1 (6.25)	–	1 (1.54)	–	–
Abdominal infection	0 (0)	1 (0.30)	–	1 (0.29)	0 (0)	1 (6.25)	–	1 (1.54)	0.166	1.000^b^
Abdominal bleeding	0 (0)	3 (0.90)	–	3 (0.86)	0 (0)	1 (6.25)	–	1 (1.54)	0.446^b^	1.000^b^
Anastomotic bleeding	0 (0)	2 (0.60)	–	2 (0.57)	0 (0)	0 (0)	–	0 (0)	–	–
intestinal obstruction	0 (0)	3 (0.90)	–	3 (0.86)	0 (0)	1 (6.25)	–	1 (1.54)	0.446^b^	1.000^b^
Respiratory complications	2 (13.33)	42 (12.61)	1.000^b^	44 (12.64)	7 (14.29)	4 (25.00)	0.543^b^	11 (16.92)	0.293^b^	<0.001
Cardiovascular complications	0 (0)	2 (0.60)	–	2 (0.57)	1 (2.04)	0 (0)	–	1 (1.54)	–	0.965^b^

Note: *B vs. D;

** *Total 1 vs. Total 2*;

b *Continuity correction; PG-SGA, patient-Generated Subjective Global Assessment*.

Meanwhile, we conducted a multivariate analysis of the risk factors that may affect postoperative complications in patients with GIST, and found that the age of patients (≥ 60 years) (*p* = 0.004, OR = 10.552, 95%CI: 2.114~52.683), intraoperative combined organ resection (*p* = 0.012, OR = 14.917, 95%CI: 1.827~121.808), and preoperative malnutrition (PG-SGA≥4) (*p* = 0.001, OR = 33.228, 95%CI: 4.060~271.970) were all independent risk factors for postoperative complications in this group of patients.

### The Relationship Between Nutritional Status and Quality of Life in GIST Patients

**Figure 11:**
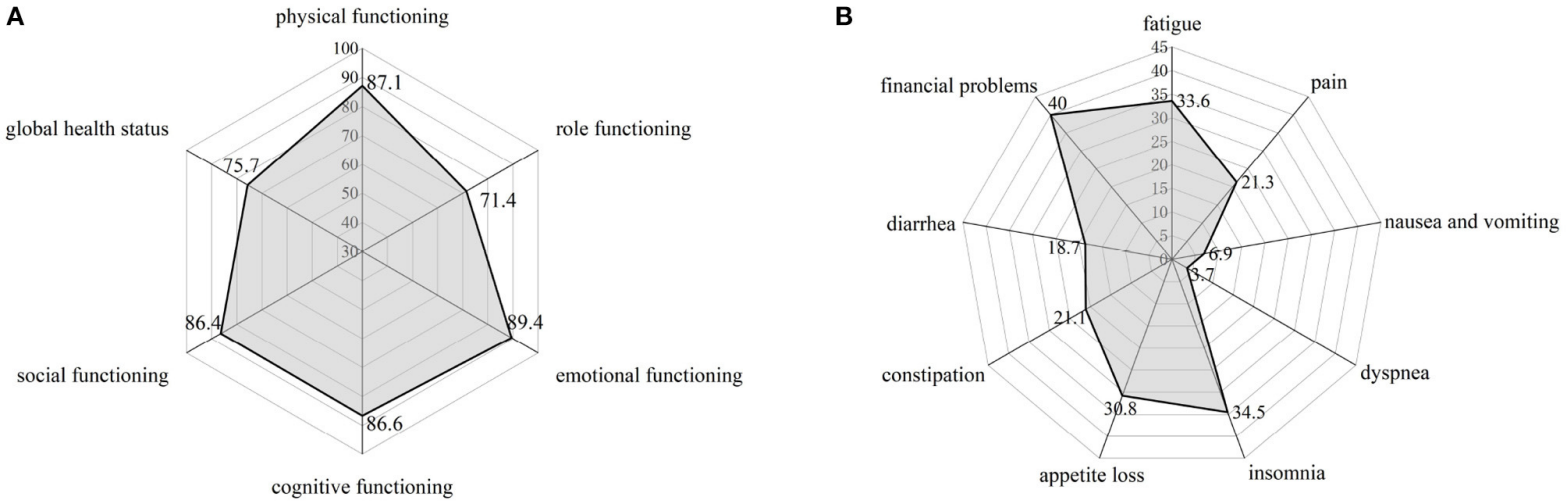
HRQoL score according to overall global health status **(A)**, sub-domains of functioning **(A)** and symptoms **(B)**.

As an indicator of quality of life, the average score of global health status of patients was 75.7. In terms of the scores of the five functional scales, the average scores of patients' social function and emotional function were the highest, but the score of role function was the lowest (Figure 11A). Among the nine medical symptoms, economic problems scored the highest, followed by insomnia and fatigue. Only a few patients reported nausea, vomiting, and dyspnea (Figure 11B).

In addition, we analyzed the correlation between the nutritional indicators of patients at discharge and the quality of life of patients, and found that the NRS2002 score (−2.769, 95%CI: −3.992~-1.546, *p* < 000.1) and PG-SGA (−4.826, 95%CI: −6.685~-1.034, *p* < 000.1) score of patients at discharge were closely related to the global health indicators of patients. Moreover, patients with good nutritional status at discharge had good HRQoL scores in other symptoms and functional scores (Table 4).

**Table 4:** Multivariable linear regression model on quality of life, symptom scales, and functional scales from the EORTC QLQ-C30.

**Factors^#^**	**Quality of life and functional scales from the EORTC QLQ-C30 questionnaire^a^**								
	**Physical functioning**	**Role functioning**	**Emotional functioning**	**Cognitive functioning**	**Social** **functioning**	**Global QoL**			
NRS2002	−1.888 (−2.915; −0.862)*	−3.885 (−5.808; −1.962)*	−2.812 (−3.945; −1.679)*	−0.851 (−2.426;0.725)	−0.970 (−2.561; 0.621)	−2.769 (−3.992; −1.546)*			
PG–SGA	−2.276 (−2.997; −1.555)*	−2.948 (−4.299; −1.597)*	−0.837 (−1.634; −0.041)*	−1.404 (−2.511; −0.297)*	−0.919 (−2.037;0.199)	−4.826 (−6.685; −1.034)*			
Weight	−0.028 (−0.178; 0.121)	−0.096 (−0.376; 0.183)	0.112 (−0.053; 0.277)	−0.243 (−0.472; −0.013)*	0.047 (−0.185; 0.278)	−0.015 (−0.193; 0.163)			
Upper arm circumference	0.104 (−0.427; 0.636)	−0.546 (−1.542; 0.450)	−0.216 (−0.803; 0.370)	0.106 (−0.709; 0.922)	0.033 (−0.791; 0.856)	−0.404 (−1.037; 0.230)			
Grip strength	0.42 2(−0.035; 0.878)	0.673 (−0.182; 1.528)	0.358 (−0.146; 0.861)	0.562 (−0.138; 1.263)	0.338 (−0.369; 1.045)	0.550 (0.007; 1.094)*			
Serum hemoglobin	0.078 (−0.050; 0.206)	0.138 (−0.102; 0.378)	0.038 (−0.103; 0.179)	0.187 (−0.010; 0.383)	0.118 (−0.081; 0.316)	0.098 (−0.055; 0.250)			
Serum albumin	0.326 (0.018; 0.635)	0.320 (−0.258; 0.898)	0.286 (−0.055; 0.627)	0.378 (−0.096; 0.852)	0.071 (−0.408; 0.549)	0.366 (−0.002; 0.734)			
Serum prealbumin	0.046 (−0.004; 0.635)	0.028 (−0.065; 0.121)	0.010 (−0.045; 0.064)	0.076 (0.000; 0.153)*	0.031 (−0.047; 0.108)	0.045 (−0.014; 0.105)			
Serum total protein	−0.050 (−0.254; 0.154)	−0.079 (−0.461; 0.304)	−0.159 (−0.384; 0.067)	−0.037 (−0.350; 0.277)	−0.237 (−0.554; 0.079)	−0.135 (−0.378; 0.109)			
**Factors** ^#^	**Symptom scales from the EORTC QLQ–C30 questionnaire^b^**								
	**Fatigue**	**Nausea /vomiting**	**Pain**	**Dyspnea**	**Insomnia**	**Appetite loss**	**Constipation**	**Diarrhea**	**Financial problem**
NRS2002	1.518 (0.441; 2.595)*	0.170 (−0.465; 0.804)	2.843 (1.260; 4.426)*	−0.173 (−0.980; 0.635)	0.449 (−1.945; 2.843)	−3.066 (−5.720; −0.412)*	0.538 (−1.487; 1.679)*	−0.641 (−2.130; 0.848)	−0.300 (−2.464; 1.864)
PG–SGA	0.971 (0.215; 1.728)*	0.265 (−0.180; 0.711)	−0.379 (−1.492; 0.733)	0.39 (−0.177; 0.958)*	0.344 (−1.338; 2.026)	0.187 (−1.678; 2.052)	−0.024 (−1.136; 1.088)	0.415 (−0.632; 1.461)	−0.057 (−1.578; 1.463)
Weight	−0.067 (−0.224; 0.090)	−0.015 (−0.108; 0.077)	−0.033 (−0.264; 0.197)	−0.098 (−0.216; 0.019)	0.241 (−0.107; 0.590)	−0.142 (−0.528; 0.245)	−0.008 (−0.238; 0.223)	0.064 (−0.153; 0.281)	−0.209 (−0.524; 0.105)
Upper arm circumference	0.144 (−0.414; 0.702)	0.006 (−0.322; 0.334)	0.063 (−0.757; 0.883)	−0.002 (−0.421; 0.416)	0.800 (−0.440; 2.039)*	1.333 (−0.041; 2.707)*	−0.366 (−1.185; 0.454)	0.358 (−0.413; 1.129)	−0.691 (−1.812; 0.429)
Grip strength	−0.145 (−0.624; 0.334)	0.101 (−0.181; 0.383)	−0.108 (−0.812; 0.596)	0.101 (−0.258; 0.460)	−0.580 (−1.645; 0.484)	−0.225 (−1.4051; 0.955)	−0.053 (−0.757; 0.650)	−0.470 (−1.132; 0.192)	0.417 (−0.546; 1.379)
Serum hemoglobin	−0.084 (−0.218; 0.051)	0.092 (0.012; 0.171)	0.026 (−0.171; 0.224)	−0.047 (−0.148; 0.054)	−0.075 (−0.374; 0.223)	−0.074 (−0.405; 0.257)	0.030 (−0.168; 0.227)	0.038 (−0.148; 0.224)	0.138 (−0.132; 0.408)
Serum albumin	−0.319 (−0.642; 0.005)	−0.105 (−0.296; 0.086)	−0.240 (−0.716; 0.236)	0.027 (−0.216; 0.270)	−0.036 (−0.756; 0.684)	−0.507 (−1.305; 0.291)	−0.076 (−0.552; 0.400)	0.246 (−0.202; 0.693)	0.441 (−0.210; 1.092)
Serum prealbumin	−0.026 (−0.079; 0.026)	0.020 (−0.010; 0.051)	−0.025 (−0.101; 0.052)	0.025 (−0.014; 0.064)	−0.035 (−0.151; 0.080)	−0.063 (−0.192; 0.065)	0.015 (−0.062; 0.092)	0.010 (−0.062; 0.082)	−0.058 (−0.162; 0.047)
Serum total protein	−0.059 (−0.273; 0.156)	−0.024 (−0.150; 0.102)	0.422 (0.107; 0.736)*	0.030 (−0.131; 0.190)	0.171 (−0.306; 0.647)	0.010 (−0.518; 0.538)	−0.080 (−0.395; 0.234)	−0.056 (−0.352; 0.241)	0.384 (−0.047; 0.814)

*Note: Scores are presented as linear regression coefficients, with 95% confidence intervals between brackets. During stepwise backward linear regression, the weakest associated variables are excluded from the model (–)*.

# *The relevant factors analyzed are all nutritional indicators measured at discharge*.

a *Higher scores represent better quality of life or functioning*.

b *Higher scores represent more symptoms*.

* *Indicate significant variables (p < 0.05)*.

## Discussion

In recent years, numerous surveys have shown that the incidence of malnutrition in cancer patients is 32%, and it is even more common in digestive tract tumors (32, 33). Currently, most studies mainly investigate the nutritional status of cancer patients during hospitalization, while few studies focus on the nutritional status of patients at discharge (34, 35). A multicenter cross-sectional survey showed that the incidence of nutritional risk (NRS 2002 ≥ 3) at discharge was significantly higher than that at hospitalization (42.82 vs. 40.12%), and the incidence of malnutrition (PG-SGA ≥ 4) was 30.47%, which was also significantly higher than 26.45% at hospitalization (36). Meanwhile, surgical resection is the most important treatment for GIST patients, but it will lead to gastrointestinal dysfunction, which will worsen the nutritional status of patients and increase the incidence of postoperative complications. Deterioration of nutritional status in discharged patients will further affect compliance with subsequent anti-tumor therapy, cause decline in quality of life, and increase readmission rate within 6 months. However, there are few reports on the changes in postoperative nutritional status of GIST patients and its impact on clinical outcomes. Our retrospective study was the first to investigate the nutritional status changes of GIST patients during perioperative period and their effects on postoperative complications and quality of life through NRS2002 nutritional risk screening combined with PG-SGA score and laboratory nutritional indicators.

By dynamically observing the nutritional status changes of GIST patients during perioperative period, we found that the body weight, grip strength, and upper arm circumference at discharge were significantly lower than those at admission, but the decrease was more obvious only in the high-risk group. This may be due to the fact that patients in the high-risk group had larger size and larger surgical trauma than those in other groups, resulting in slow recovery of gastrointestinal function and insufficient postoperative nutritional intake. Moreover, we also found that serum albumin, prealbumin, and total protein were significantly lower than those at admission, which may be related to the increase of protein catabolism caused by traumatic stress stimulation after surgical treatment, thus causing the deterioration of nutritional status. This is similar to the results of Zhu et al. (36). Furthermore, our results also discovered that nutritional risk and malnutrition were common in GIST patients during perioperative period, especially when patients were discharged after surgery. Through NRS2002 screening and PG-SGA assessment within 24 h after admission and 24 h before discharge, it was found that the incidence of nutritional risk and malnutrition at discharge was 27.60 and 15.73%, respectively, which was significantly higher than 46.73 and 37.29% at admission. Interestingly, our further stratified analysis found that risk of malnutrition in patients in the high-risk group and in patients which located in the mesentery was significantly higher than that in other groups. This suggests that we need to pay more attention to the nutritional status changes, nutritional monitoring, and treatment of GIST patients during perioperative period, especially when the patients are discharged.

We further analyzed the effect of perioperative nutritional status on clinical outcomes of GIST patients, and found that the incidence of surgical-related complications in patients with malnutrition (PG-SGA score ≥ 4) (29.23%) was significantly higher than that in patients without malnutrition (18.10%). In addition, the study also showed that patients with nutritional risk had lower incidence of complications than patients without nutritional support (*p* = 0.006), but patients without nutritional risk could not benefit from nutritional support (*p* > 0.05). Moreover, we found that preoperative malnutrition (PG-SGA score ≥ 4) was one of the independent risk factors for postoperative complications. Numerous studies have also confirmed that the incidence of postoperative complications in patients receiving preoperative nutritional support is significantly reduced. Jie et al. found that for patients with PG-SGA ≥ 4, 50% of patients without nutritional support had complications, but the incidence of complications in patients receiving nutritional support was reduced to 26% (37, 38). The results of this study are consistent with the above studies, which suggests that it is necessary to provide preoperative nutritional support for patients with malnutrition, so as to reduce the incidence of postoperative complications.

This retrospective study was the first to investigate the relationship between postoperative quality of life and nutritional status of GIST patients at discharge. Interestingly, we found that the nutritional status of patients at discharge is closely related to the quality of life. Most importantly, the NRS2002 score and PG-SGA score of patients at discharge were closely related to the global health indicators of patients. These findings have also been supported by other studies, which also found the relationship between nutritional status and quality of life in cancer patients. Zhang et al. found that the nutritional status of patients with gastrointestinal cancer determines the quality of life during subsequent treatment (39). Moreover, other scholars also found that the nutritional status of patients may be a decisive factor affecting the quality of life of patients with advanced cancer after discharge, especially in patients with upper gastrointestinal cancer (40, 41). In view of these results, we speculated that the nutritional status of patients at discharge, especially NRS2002 score and PG-SGA score may play a more important role in evaluating the quality of life of patients with GIST after surgery.

There are still some limitations of this study that need to be addressed. First, this study is a single-center retrospective study with limited number of cases. Second, we only investigated the postoperative complications and the quality of life at 1 month after surgery, but lacked the long-term dynamic assessment of quality of life after surgery. Third, we did not follow up the survival status of patients, so it was impossible to assess the impact of postoperative nutritional status on the long-term prognosis of patients. Therefore, it is necessary to further carry out multi-center prospective studies to assess the impact of perioperative nutritional status changes in GIST patients on long-term clinical outcomes such as prognosis, quality of life, and subsequent treatment tolerance.

## Conclusion

In this study, NRS2002 nutritional risk screening combined with PG-SGA nutritional assessment and other nutritional related indicators were used for the first time to dynamically assess the nutritional status changes of GIST patients during perioperative period. Studies have shown that the proportion of nutritional risk (27.60%) and malnutrition (15.73%) in GIST patients at admission is high, but the nutritional status is further deteriorated at discharge, and the nutritional risk and malnutrition rates are 46.73 and 37.29%, respectively. Most importantly, poor perioperative nutritional status is also closely related to poor clinical outcomes. Therefore, NRS2002 nutritional screening, PG-SGA nutritional assessment and other nutrition-related indicators (weight, grip strength, upper arm circumference, serum hemoglobin, albumin, prealbumin, and total protein) should be dynamically monitored in patients with GIST during perioperative period, and necessary nutritional support should be given to patients with malnutrition.

## Data Availability Statement

The raw data supporting the conclusions of this article will be made available by the authors, without undue reservation.

## Ethics Statement

The study design was approved by the Ethics Committee of the Fourth Hospital of Hebei Medical University (Approval Number: 2018088). The patients/participants provided their written informed consent to participate in this study.

## Author Contributions

QZ: conception and design and administrative support. PD, PY, YT, HG, YoL, CS, ZZ, DW, XZ, BT, and YuL: provision of study materials or patients. PD, PY, YT, HG, and YaL: collection and assembly of data. PD, HG, and CS: data analysis and interpretation. All authors contributed to the article and approved the submitted version.

## Funding

This work was supported by the Cultivating Outstanding Talents Project of Hebei Provincial Government Fund (No. 2019012), Hebei public health committee county-level public hospitals suitable health technology promotion and storage project (No. 2019024), Hebei Medical University Education and Teaching Research Project (Nos. 2020CGPY-12 and 2020CHYB-23), and Hebei University Science and Technology Research Project (No. ZD2019139).

## Conflict of Interest

The authors declare that the research was conducted in the absence of any commercial or financial relationships that could be construed as a potential conflict of interest.

## Publisher's Note

All claims expressed in this article are solely those of the authors and do not necessarily represent those of their affiliated organizations, or those of the publisher, the editors and the reviewers. Any product that may be evaluated in this article, or claim that may be made by its manufacturer, is not guaranteed or endorsed by the publisher.
